# Secretory expression of amylosucrase in *Bacillus licheniformis* through twin-arginine translocation pathway

**DOI:** 10.1093/jimb/kuae004

**Published:** 2024-01-22

**Authors:** Caizhe Wang, Dandan Niu, Nokuthula Peace Mchunu, Meng Zhang, Suren Singh, Zhengxiang Wang

**Affiliations:** Department of Biological Chemical Engineering, College of Chemical Engineering and Materials Science, Tianjin University of Science and Technology, Tianjin 300457, China; Department of Biological Chemical Engineering, College of Chemical Engineering and Materials Science, Tianjin University of Science and Technology, Tianjin 300457, China; National Research Foundation, PO Box 2600 Pretoria 0001, South Africa; School of Life Science, University of KwaZulu Natal, Durban 4000, South Africa; Department of Biological Chemical Engineering, College of Chemical Engineering and Materials Science, Tianjin University of Science and Technology, Tianjin 300457, China; Department of Biotechnology and Food Science, Faculty of Applied Sciences, Durban University of Technology, PO Box 1334, Durban 4001, South Africa; Department of Biological Chemical Engineering, College of Chemical Engineering and Materials Science, Tianjin University of Science and Technology, Tianjin 300457, China; Tianjin Key Laboratory of Industrial Microbiology, Tianjin 300457, China

**Keywords:** Amylosucrase, Secretory expression, Twin-arginine translocation pathway, *Bacillus licheniformis*

## Abstract

Amylosucrase (EC 2.4.1.4) is a versatile enzyme with significant potential in biotechnology and food production. To facilitate its efficient preparation, a novel expression strategy was implemented in *Bacillus licheniformis* for the secretory expression of *Neisseria polysaccharea* amylosucrase (NpAS). The host strain *B. licheniformis* CBBD302 underwent genetic modification through the deletion of *sacB*, a gene responsible for encoding levansucrase that synthesizes extracellular levan from sucrose, resulting in a levan-deficient strain, *B. licheniformis* CBBD302B. *Neisseria polysaccharea* amylosucrase was successfully expressed in *B. licheniformis* CBBD302B using the highly efficient Sec-type signal peptide S*_amyL_*, but its extracellular translocation was unsuccessful. Consequently, the expression of NpAS via the twin-arginine translocation (TAT) pathway was investigated using the signal peptide S*_glmU_*. The study revealed that NpAS could be effectively translocated extracellularly through the TAT pathway, with the signal peptide S*_glmU_* facilitating the process. Remarkably, 62.81% of the total expressed activity was detected in the medium. This study marks the first successful secretory expression of NpAS in *Bacillus* species host cells, establishing a foundation for its future efficient production.

**One-Sentence Summary:**

Amylosucrase was secreted in *Bacillus licheniformis* via the twin-arginine translocation pathway.

## Graphical Abstract

**Graphical Abstract:**
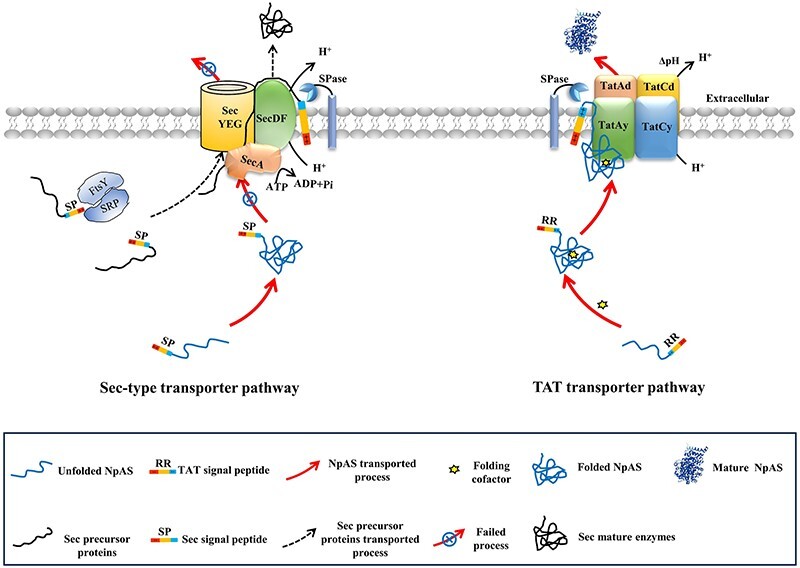

## Introduction

Amylosucrase (AS, EC 2.4.1.4), first discovered in *Neisseria perflava* (Hehre & Hamilton, 1948), is a glucosyltransferase enzyme from the Glycoside hydrolase (GH) family 13 (Moulis et al., 2016). Amylosucrase is unique for its ability to use sucrose, its natural substrate, to create amylose-type polymers. These polymers are notable for their helical structure and controlled regularity, making them functional supramolecular materials (Putseys et al., 2010; Seung, 2020). Unlike other amylopolysaccharide synthases, AS polymerizes without the need for α-D-glucosyl nucleoside diphosphate glucosyl donors, such as adenosine diphosphate (ADP)- or uridine diphosphate (UDP)-glucose (Seo et al., 2020). This characteristic has established AS as a valuable tool in industry for synthesizing or modifying a diverse array of polysaccharides and their derivatives. When reacting with sucrose, AS can be used to design and create amylodextrins with specific morphologies, structures, physicochemical properties, and degrees of polymerization (Potocki-Veronese et al., 2005). Additionally, AS can catalyze the isomerization of sucrose to produce turanose, a functional sweetener, especially at high sucrose concentrations with added fructose as a modulator (Park et al., 2016). In combination with maltooligosyltrehalose synthase and maltooligosyltrehalose trehalohydrolase, AS facilitates the one-pot bioconversion of sucrose to trehalose (Jung et al., 2013). Furthermore, in the presence of sucrose and alternative non-natural glycosyl acceptors, AS can transfer glucose from sucrose to these acceptors, leading to the synthesis of novel compounds. Examples include carbohydrate-based dendritic nanoparticles (Putaux et al., 2006; Lee et al., 2022;) and baicalein-6-glucoside, which boasts enhanced stability, water solubility, and improved physiological properties (Kim et al., 2014), unusual quercetin diglucosides and isoquercitrin glucosides (Rha et al., 2020a, 2020b) are among the compounds whose bioavailability can be enhanced through glycosylation by AS (Moon et al., 2021). This enzyme can also catalyze the formation of both α and β anomers of glycosides in triterpenoids from the medicinal fungus *Ganoderma lucidum* (Wu et al., 2022), and improve the solubility of puerarin (Ding et al., 2022).

The exceptional and distinctive application properties of AS have spurred significant interest in the gene mining, cloning, and expression of novel AS enzymes. Since the initial biochemical identification of extracellular AS in *Neisseria polysaccharea* in 1983 (Riou et al., 1983), it has been cloned, heterologously expressed, and characterized in *Escherichia coli* (Büttcher et al., 1997). Numerous other novel ASs have since been gene cloned and/or functionally identified in a variety of organisms, including *Deinococcus radiodurans* (Pizzut-Serin et al., 2005), *Deinococcus geothermalis* (Emond et al., 2008), *Alteromonas macleodii* (Ha et al., 2009), *Arthrobacter chlorophenolicus* (Seo et al., 2012), *Synechococcus* sp. (Perez-Cenci & Salerno, 2014), *Cellulomonas carboniz* (Wang et al., 2017), and *Bifidobacterium thermophilum* (Choi et al., 2019). However, these identified AS enzymes are often fused with a glutathione-S-transferase or histidine tag and are expressed and prepared in inclusion bodies in *E. coli*. Despite extensive improvements in the expression efficiency of foreign proteins in *E. coli* (Schneider et al., 2011), the practicality of low-cost and large-scale enzyme preparation is hindered by low expression levels, the complexity of downstream processing, and potential endotoxin contamination. Heterologous expression in more efficient host systems, such as *Bacillus subtilis*, is necessary but has yet to be achieved for reasons that remain unclear (Kim et al., 2019). This motivates us to devise a novel routine for the high-efficiency preparation of AS by employing alternative expression systems.

The current studies are designed to investigate the potential for secretory expression of NpAS in a food-grade *B. licheniformis* expression system. By examining peptide translocation pathways, we explored both the general transport (Sec) pathway and the twin-arginine translocation (TAT) pathway to determine if NpAS secretion is feasible through both S*_amyL_* (a Sec signal peptide) and S*_glmU_* (a TAT signal peptide). We conducted a comparative analysis of the main forms and functions of secretory recombinant NpAS and re-verified them accordingly. Establishing this new expression routine for NpAS could provide the groundwork for its future large-scale and efficient production.

## Materials and Methods

### Strains, Plasmids, and Propagation Conditions

The strains and plasmids used in this study are listed in Table 1. *Escherichia coli* JM109 was used as the host for gene cloning and plasmid construction. *Bacillus licheniformis* CBBD302 (Niu et al., 2009) was employed as the parent strain for new host cell development. Temperature-sensitive replicative shuttle plasmid pUB-EX (Shen et al., 2022) was applied for gene deletion in *B. licheniformis*. pHY-WZX (Niu & Wang, 2007) harboring signal peptide S*_amyL_* was used for mediating the secretion and expression of NpAS or as a parent plasmid for new plasmid construction. All strains were cultivated at 37°C in Luria–Bertani (LB) medium (1% tryptone, 0.5% yeast extract, and 1% NaCl). Luria–Bertani plates were prepared by adding 1.5% agar powder to the LB medium. When necessary, 20 μg/ml kanamycin was supplemented.

**Table 1.** Strains and plasmids used in this study

**Strain/Plasmid**	**Characteristics**	**Source**
**Strain**		
*E. coli* JM109	*endA1, recA1, gyrA96, thi, hsdR17, rel*A1, *sup*E44, λ^−^, Δ(*lac*-*proAB*), [F´, *traD36, proAB, laqI^q^Z*ΔM15]	Lab stock
*B. subtilis* WB600	*B. subtilis* 168, Δ*nprE*, Δ*aprA*, Δ*epr*, Δ*bpf*, Δ*mpr*, Δ*nprB*	Lab stock
*B. licheniformis* CBBD302	Host cell for gene expression	(Niu et al., 2009)
*B. licheniformis* CBBD302B	*B. licheniformis* CBBD302, Δ*sacB*	This study
BL-AS1	*B. licheniformis* CBBD302, Δ*sacB*, harboring pHY-AS1	This study
BL-1.0AS1	*B. licheniformis* CBBD302, Δ*sacB*, harboring pTAT1.0-AS1	This study
**Plasmid**		
pHY-WZX	Harboring S*_amyL_* from *B. licheniformis*, Tet^R^, Ap^R^, Km^R^, expression vector	(Niu & Wang, 2007)
pUB-EX	Km^R^, thermosensitive plasmid, harboring the expression cassette of pHY-WZX	(Shen et al., 2022)
pTAT1.0	Replaced S*_amyL_* of pHY-WZX by S*_glmU_*, Km^R^	This study
pHY-AS1	Harboring *npas* gene in pHY-WZX, Km^R^	This study
pTAT1.0-AS1	Harboring *npas* gene in pTAT1.0, Km^R^	This study
pUB-*sacB'*	Harboring *B. licheniformis sacB* deletion cassette, Km^R^	This study
pUC57-*npas*	Chemically synthesized *npas* cloned in pUC57	This study

### Genetic Manipulation

The conventional laboratory DNA manipulations, including chromosomal DNA isolation, polymerase chain reaction (PCR), plasmid mini-preparation, DNA restriction endonuclease digestion, ligation, and transformation of *E. coli* were performed according to the established protocols (Sambrook & Russel, 2001). The primers used in this study are listed in Table 2. The full nucleotide sequence of an ASase-encoding gene *npas* from *N. polysaccharea* (locus_tag: AJ011781.1) was codon-optimized and chemically synthesized by Sangon Biotech Co., Ltd., China, for expression in *B. licheniformis*.

**Table 2.** Nucleotide sequence of primers

**Oligonucleotides**	**Nucleotide sequence (5′ to 3′)** *
**For plasmid construction**	
318-R	TCGGTTCCCTCCTCACTTTTC
318-F	TTACAGGATCCTCTAGAATTCCCG
Glu-F	ATGGATAAAAGGGATAATGGAGG
Glu-R	CCTGGATCCAGGATGAAGAACTTTATATAGC
**For gene deletion**	
Amys113-F	TATGGATCCATGCTGACGC
Amys113-R	TTAGGCGATTTCCAGCCAC
LSC-up1	TCAAAGAAGCCGTCCAAGA
LSC-up2	CCCTGTTCAAGGATGCTGTCCCGGGCATTGTCCTGAAGAACAG
LSC-dn1	CTGTTCTTCAGGACAATGCCCGGGACAGCATCCTTGAACAGGG
LSC-dn2	AGAGGATCCTCTTCCTTCCCGTCATTG
UP-LSCR	TCAGCATGTCATGTCTTGTGATAT
DN-LSCF	CCAGGATTACAATGACATCACG
T2-MCS2F	GCAAGCAGCAGATTACGC
T2-MCS2R	ATGTGATAACTCGGCGTA
LSC-F	GAAACACCTGGAATCACAATGG
LSC-R	CATCCTGTTCATCCCAGATCAC

*Underlined represented *Bam*HI or *Sma*I restriction site.

The upstream and downstream fragments flanked *sacB* were amplified by PCR with primers LSC-up1/LSC-up2 and LSC-dn1/LSC-dn2, respectively, using the chromosomal DNA of *B. licheniformis* CBBD302 as template. The amplified upstream and downstream fragments were then mixed and used as the template for the preparation of the deletion cassette, *sacB*’, with primer LSC-up1/LSC-dn2. It was then purified and digested by *Bam*HI, and cloned into *Bam*HI and *Sma*I sites of pUB-EX (Shen et al., 2022), resulting in pUB-sacB’. This construct was then transformed into *B. licheniformis* CBBD302. The *sacB* deletion mutant was obtained by the homologous recombination-mediated double-exchange method (Zhou et al., 2020) and confirmed by diagnosis PCR. The levan-deficient strain was further confirmed by cultivating it on an LB plate containing 5% sucrose at 37°C for 24 hr to observe the levan formation or inoculated at a 10% dose in 50 ml LB liquid medium (containing 5% sucrose) at 37°C and 200 rpm for 48 hr. After fermentation, the cultures were centrifuged at 8,000 × *g* for 15 min. The supernatants were dealt with two volumes of chilled ethanol to precipitate polymers overnight at −20°C followed by centrifugation at 8,000 × *g* for 15 min, and dried by desiccation in vacuo. Polymer pellets were then re-dissolved in 50 ml of warm ddH_2_O and determined by quantifying the carbohydrate content as fructose equivalents using the phenol-sulfuric acid method (Liu et al., 2010). Briefly, a standard curve between the concentration of fructose and the absorbance at OD_490_ was first established after incubation with sulfuric acid and phenol. Then, the content of levan could be measured according to the curve.

### Strain Development

The DNA fragment encoding the signal peptide S*_glmU_* (Niu et al., 2019) was amplified by using *B. licheniformis* CBBD302 genomic DNA as template with primers Glu-F and Glu-R. The S*_amyL_* fragment in pHY-WZX was removed by reverse PCR with primers 318-F and 318-R, followed by digestion with *Bam*HI and ligation with *Bam*HI-digested S*_glmU_* fragment to create the new plasmid pTAT1.0.

The whole-length gene for NpAS (named *npas*) amplified pUC57-*npas* by PCR with primers Amy113-F and Amy113-R. The recovered *npas* fragment was digested by *Bam*HI and cloned into the *Bam*HI and *Sma*I sites of either pHY-WZX or the newly constructed pTAT1.0. The recombinant plasmids were then transformed into *B. licheniformis* by electroporation and the resulting transformants were selected for subsequent studies.

### NpAS Expression in *B. Licheniformis*

#### Semi-quantitative Analysis of Enzyme Expression Level on Plates

Recombinant strains were cultivated to a cell density (OD_600_) of 3.0–3.5 in 50 ml LB medium at 37°C for 12–16 hr. One μl of the culture broth was dotted on an LB plate (complemented with 5% sucrose). The plates were incubated at 37°C for 12 hr, 24 hr, or 36 hr, and the yields of AS produced by strains were evaluated using iodine vapor staining. The stained area of colonies or halo-surrounded colonies was used as a parameter for semi-quantitative analysis of the enzyme production capacity (Büttcher et al., 1997).

#### Shaking Flask Fermentation of NpAS

Shake flask fermentation was performed with some modifications as described in the previous study (Niu et al., 2009). Recombinant colonies were inoculated into a 50 ml of LB liquid medium with 20 μg/ml kanamycin and cultured at 200 rpm and 37°C for 12–16 hr. Preparation of NpAS from recombinant strains was carried out with a 10% dose using shake flask fermentation in a 250 ml Erlenmeyer flask with a working volume of 50 ml of LB supplemented with lactose at a final concentration of 1% at 37°C and 220 rpm for 84 hr. The cell mass of recombinant NpAS expressed in the levan­deficient mutant was determined by regularly measuring the optical density of the cultures at 600 nm. The dry cell weight (DCW) was measured as follows. A 50 ml of fermentation culture was collected and centrifuged at 8,000 × *g* at 4°C for 20 min and washed 3 times with 0.9% NaCl solution. The cell pellets were dried at 55°C to constant weight and recorded.

#### Impact of Cell Wall-weakening Reagents on NpAS Expression

When necessary, glycine, sodium penicillin G, Sodium dodecyl sulfate (SDS), or Triton X-100 were selected as the cell wall-weakening reagents. Different concentrations of reagents, including glycine (0.25, 0.5, 1.0, 1.5, 2.0, 2.5, and 3.0%, w/v, final concentration), sodium penicillin G (40, 80 160, 240, and 320 mg/ml), SDS (0.01, 0.025, 0.05, 0.10, and 0.20%, w/v), and Triton X-100 (0.25, 0.5, 1.0, 1.5, and 2.0%, w/v) were added to the shake flask fermentation at 60 hr and then incubated for an additional 24 hr. Post-incubation, the intracellular and extracellular enzyme activity of NpAS and cell mass of recombinant *B. licheniformis* were determined.

### Enzyme Purification and Activity Assay

#### Sample Preparation

The culture broth was centrifuged at 8,000 × *g* at 4°C for 20 min, the supernatant was used for further study. The cell pellet was washed twice with ddH_2_O and resuspended in 50 mM Tris-HCl (pH 7.0) and lysed with ultrasonication (SCIENTZ-IID, Scientz, Ningbo, Zhejiang, China; output power 400 W, 20 times for 3 s, constant duty) in an ice bath. The disrupted cells were centrifuged (8,000 × *g* at 4°C for 20 min) and the resulting supernatant was filtered through a 0.45 μm filter used for further study.

#### Isolation and Purification

The broth with recombinant NpAS was precipitated using 40–70% saturated ammonium sulfate solution. The precipitants were then collected by centrifugation at 8,000 × *g* and 4°C for 20 min. The pellets were collected and resuspended in 50 mM Tris–HCl buffer (pH 7.0) and dialyzed against the same buffer. Subsequently, the NpAS was purified by an ÄKTA Pure system with a Sephadex G-100 (10 × 500 mm) column (Cytiva, Sweden) by eluting with 50 mM phosphate buffer (pH 7.0) at a flow rate of 0.5 ml/min. The molecular weight of NpAS was estimated based on SDS-PAGE (Zhu & Wang, 1994), selecting 10% (w/v) running gel and 5% (w/v) stacking gel. Protein concentration was measured using the Micro Bradford method (Bradford, 1976) using bovine serum albumin FV (Roche Diagnostics GmbH, Germany) as the standard.

#### Enzyme Activity Assay

The NpAS activity assay (total 0.4 ml) was conducted in 50 mM Tris-HCl buffer (pH 7.0) with 0.1 mol/l sucrose as a substrate at 35°C for 30 min. An inactivated enzyme sample, boiled for 10 min, served as control. After the reaction, the amount of released fructose was measured by the dinitrosalicylic acid (DNS) method (Zhu & Wang, 1994) with fructose as a standard. Briefly, the 0.4 ml reaction mixture was terminated by adding 0.6 ml of DNS solution, followed by heating at 100°C for 7 min and cooling on ice. Absorbance was measured at 550 nm. One unit of NpAS activity is defined as the amount of enzyme releasing 1.0 μmol of fructose per minute under the assay conditions (Kim et al., 2019).

#### Carbohydrate Profiles in NpAS-catalytic Reaction Mixture

The enzyme reaction was performed with 75 mM sucrose and 0.1 u/ml of pure NpAS at 35°C and pH 7.0 reaction for 6 hr, 24 hr, 48 hr, and 120 hr. Post-reaction, the collected samples were separated by centrifugation (8,000 × *g*, 4°C). The water-insoluble fractions were subsequently stained with an iodine solution to identify the generated amylose polymers according to the method described (van der Veen et al., 2006). Soluble products were quantified using high performance liquid chromatography (HPLC) on an Alltech Prevail Carbohydrate ES 5 u column (4.6 mm ID × 250 mm, 5 μm) with 65% (v/v) acetonitrile as mobile phase at a flow rate of 1 ml/min at 35°C. The reference standard for glucose, fructose, sucrose, and maltooligosaccharides was purchased from Jiangsu Ruiyang Biotech Co., Ltd., China. The reference standard for turanose was purchased from Sangon Biotech (Shanghai) Co., Ltd.

## Result and Discussion

### Construction of Levan-Deficient *B. Licheniformis*

Almost all isolates of *B. licheniformis* have been verified to produce extracellular levan polysaccharide, a naturally occurring homopolymer fructan from sucrose catalyzed by levansucrase (EC 2.4.1.10) (Liu et al., 2010; Nakapong et al., 2013). This enzyme is an extracellular enzyme encoded by gene *sacB* in *B. licheniformis* (He et al., 2018). It catalyzes sucrose hydrolysis and directly transferes the fructosyl moiety to another sucrose acceptor, resulting in levan formation and the release of free glucose (Klaewkla et al., 2020). The existence of levansucrase may complicate the purification and subsequent applications of NpAS due to they share the same substrate (sucrose) but yield different products. Consequently, it is crucial to prevent levansucrase production in the expression host by deleting the *sacB* gene.

**Fig. 1.**
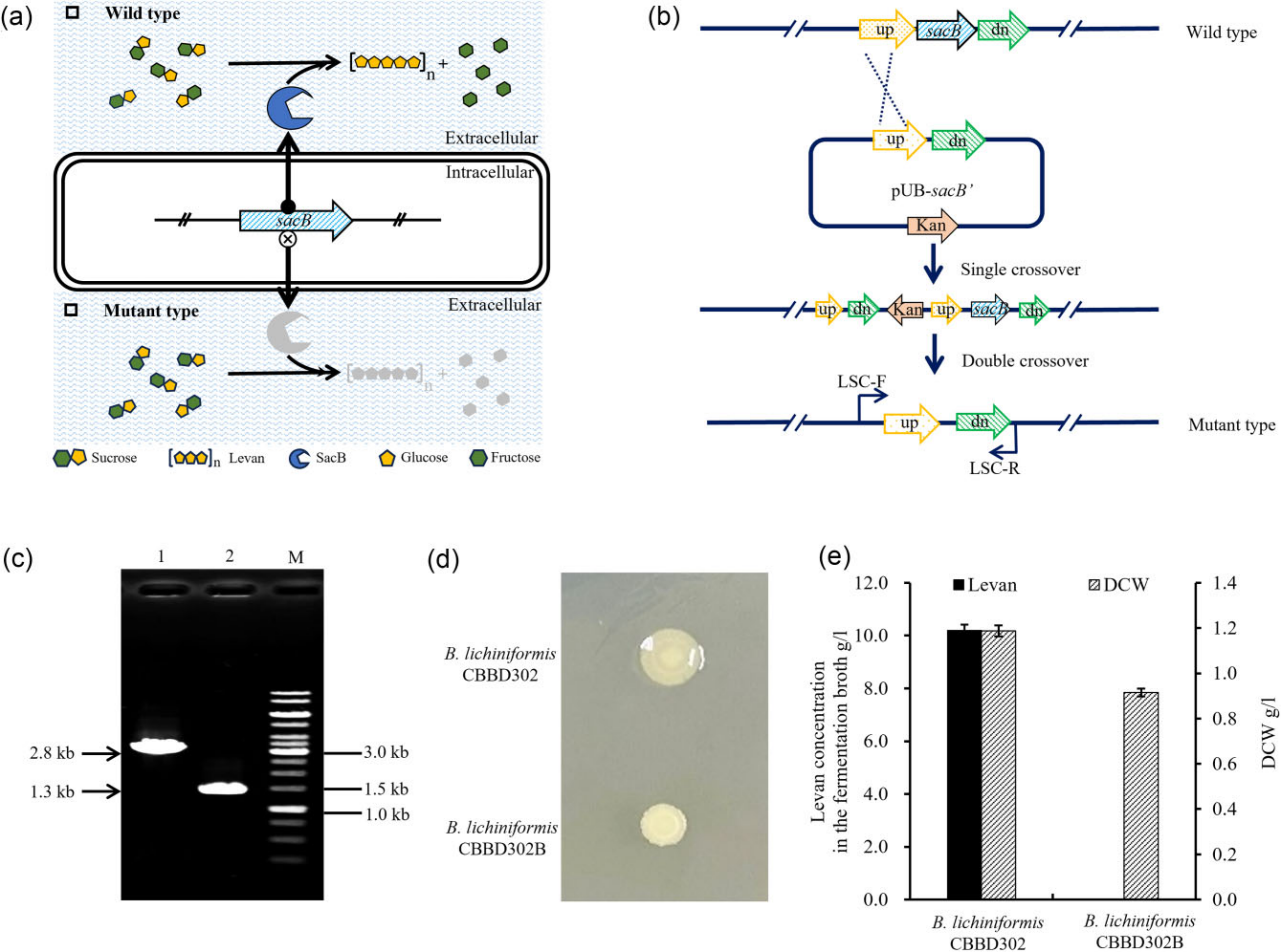
Verification and characterizations of *sacB* deletion in *B. licheniformis*. (a) Schematic illustration representing the results of *sacB* deletion in *B. licheniformis* CBBD302. (b) Design of the primers used in the verification of the double-crossover mutant when screening the integrons of *sacB* cassette. LSC-F and LSC-R were primer pairs used for PCR validation and pUB-*sacB’* was used for the deletion of *sacB*. (c) Verification of the *sacB* deleting strains by colony PCR. (M) DNA molecular weight marker; lane 1: PCR product of parent strain with an amplified fragment of 2.8 kb; lane 2: PCR product of mutant *B. licheniformis* CBBD302 with an amplified fragment of 1.3 kb. (d) Levan forming assay on LB plate. The levan-deficient strain and parent strain were cultured on an LB plate (containing 5% sucrose) at 37°C for 24 hr to observe the formation of levan. (e) Levan yield by *B. licheniformis* CBBD302 and *B. licheniformis* CBBD302B in 250 ml Erlenmeyer flask with a working volume of 50 ml. The incubation was carried out at 37°C and 200 rpm for 48 hr. The samples were collected and the DCW and levan contents were determined. Values are means of three replications ± standard deviation.

The *sacB* gene, encoding levansucrase for the synthesis of extracellular levan (Fig. 1a), was deleted (Fig. 1b) from the parental *B. licheniformis* CBBD302 strain. The resultant *sacB* deletion mutant was confirmed by diagnostic PCR (Fig. 1c) and redesignated as *B. licheniformis* CBBD302B. After a period of incubation on LB plates with sucrose, the colonies exhibited a rough, dry, and non-mucoid appearance, which was different from that of the parent strain (a smooth, humid, and mucoid colony) (Fig. 1d). This changed morphology was likely due to the loss of levan synthesis in *B. licheniformis* CBBD302B. Subsequently, *B. licheniformis* CBBD302B was cultivated by shake flask fermentation. The concentration of levan in the broth was quantified and the findings are summarized in Fig. 1e. Clearly, *B. licheniformis* CBBD302B revealed no detectable levan, while the parent strain could produce 10.2 g/l of levan under identical conditions. The results suggest that the production of levan in *B. licheniformis* CBBD302 was governed by a single *sacB* gene product, rendering *B. licheniformis* CBBD302B deficient in levan.

### Sec Translation Pathway in *B. Licheniformis* Was Unable to Mediate the Secretion of NpAS

Many mechanisms and biochemical types are involved in protein translocation with different selections or affinities (Anne et al., 2017). Of which, the Sec pathway is ubiquitous and essential to directly export a majority number of proteins under their unfolded forms through a well-organized cargo across the cell membrane and cell wall in a process of either co-translational or post-translational (Tsirigotaki et al., 2017). The Sec pathway can overexpress and efficiently prepare many kinds of industrially important enzymes (Niu et al., 2009, 2022; Shen et al., 2022). Therefore, we tried to examine the possibility of the Sec pathway in *B. licheniformis* for secretory expression of NpAS.

**Fig. 2.**
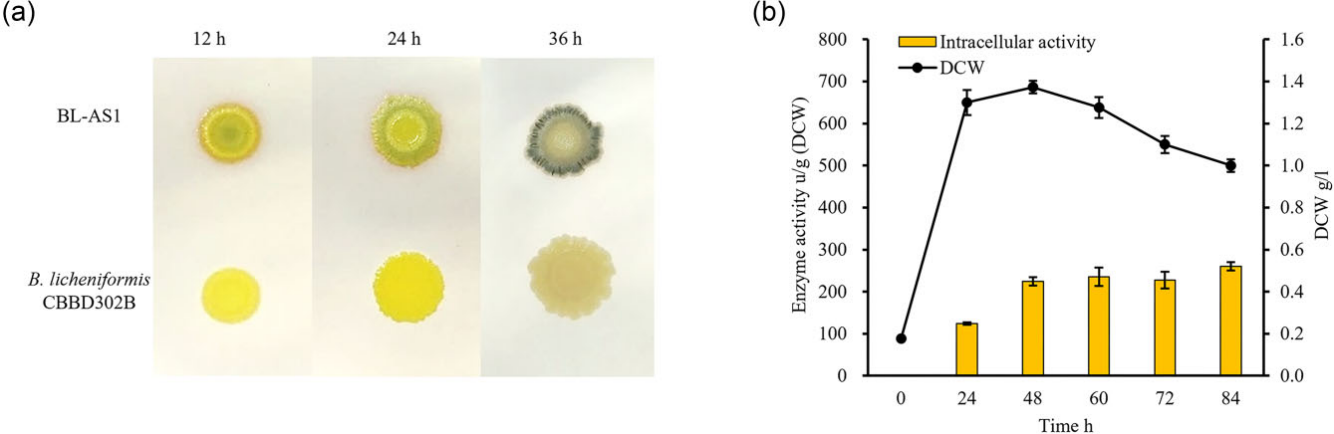
The expression of NpAS by Sec translation pathway in *B. licheniformis*. (a) After cultured for 12 hr, 24 hr, and 36 hr, amylose forming assay on an LB plate (containing 5% sucrose) by BL-AS1 and *B. licheniformis* CBBD302B stained with iodine vapor. (b) NpAS yield by BL-AS1 in 250 ml Erlenmeyer flask with a working volume of 50 ml. The incubation was carried out at 37°C and 220 rpm for 84 hr. The samples were collected and their NpAS activity and DCW were determined. The error bar indicates the standard deviation from three parallel experiments.

The *npas* gene encoding the matured peptide of NpAS (Büttcher et al., 1997) was codon-optimized and chemically synthesized. It was then cloned in-frame after the S*_amyL_* in the expression plasmid pHY-WZX (Niu & Wang, 2007). The resulting recombinant plasmid pHY-AS1 was transformed into *B. licheniformis* CBBD302B, yielding a recombinant strain BL-AS1 (*B. licheniformis* CBBD302B harboring pHY-AS1). Clear and amylose-specific materials formed in these cells after cultivating on sucrose-containing plates and an altered iodine staining pattern changed from red (12 hr) to blue (36 hr) (Fig. 2a), which indicated that the recombinant strain BL-AS1 functionally expressed NpAS. Consequently, the NpAS yields were further examined in shake-flask fermentation experiments. Contrary to expectations, no detectable NpAS activity in the broth was found, although there existed an obvious NpAS activity inside the cells (Fig. 2b). These results strongly indicated that NpAS molecules were unable to be translocated through the Sec pathway in *B. licheniformis*. The possible reason is that the NpAS peptide may fold to form well-folded NpAS too fast to be recognized and handled by Sec translocase in *B. licheniformis* (Denks et al., 2014). The results illustrated that NpAS was actively expressed in *B. licheniformis*, however, it could not be translocated extracellularly through the Sec pathway.

Alternatively, the TAT pathway, another well-characterized secretion pathway, provides a fascinating alternative for protein secretion when proper folding occurred within the cytoplasm prior to secretion (Berks, 2015). Proteins such as GFP (Albiniak et al., 2013), protein glutaminase (Niu et al., 2019), disulfide bond‐containing protein (Arauzo-Aguilera et al., 2023), and laccases (Valimets et al., 2023) have been successfully secreted using the TAT pathway. Next, we will consider the possibility of NpAS being targeted for export via the TAT pathway.

### The TAT Translation Pathway in *B. Licheniformis* Can Mediate the Secretion of NpAS

**Fig. 3.**
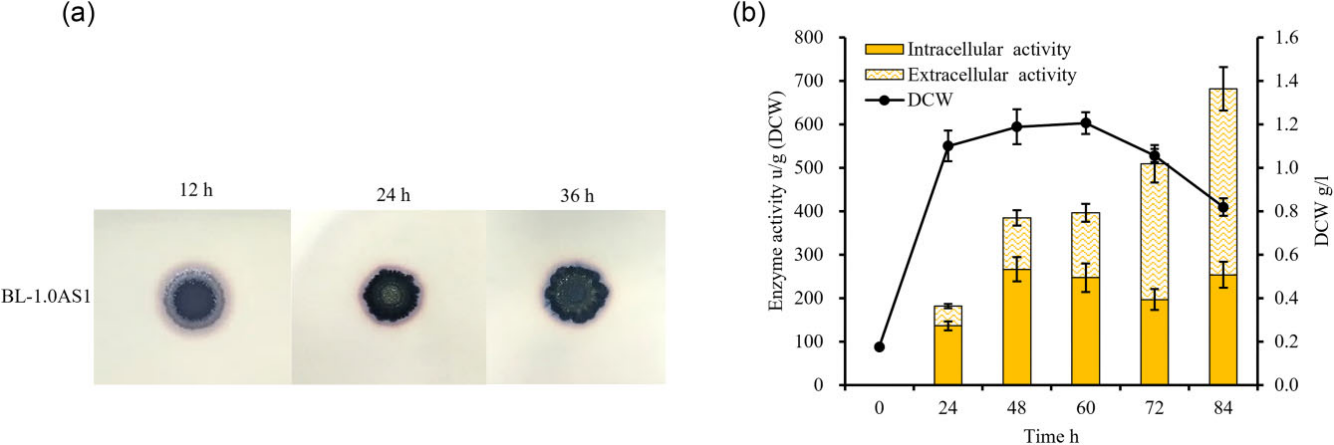
The expression of NpAS by TAT translation pathway in *B. licheniformis*. (a) After cultured for 12 hr, 24 hr, and 36 hr, amylose forming assay on LB plate (containing 5% sucrose) by BL-1.0AS1 stained with iodine vapor. (b) NpAS yield by BL-1.0AS1 in 250 ml Erlenmeyer flask with a working volume of 50 ml. The incubation was carried out at 37°C and 220 rpm for 84 hr. The samples were collected, and their NpAS activity and DCW were determined. The error bar indicates the standard deviation from three parallel experiments.

A typical TAT translocation pathway was found by mining the genome of *B. licheniformis*. A novel expression plasmid (designated as pTAT1.0) was constructed by replacing the nucleotide sequence coding for AmyL signal peptide in the plasmid pHY-WZX (Niu & Wang, 2007) with the nucleotide sequences encoding for GlmU twin-arginine signal peptide (S*_glmU_*) (Niu et al., 2019). Subsequently, the *npas* gene was cloned in-frame after S*_glmU_*, yielding a recombination plasmid pTAT1.0-AS1. A recombinant strain BL-1.0AS1 was developed by transforming pTAT1.0-AS1 into *B. licheniformis* CBBD302B and cultivated on LB plates (containing 5% sucrose) for 36 hr. Post-incubation, a clear and amylose-specific halo was formed in the periphery of the colonies (Fig. 3a), which indicated that NpAS was functionally expressed and may be extracellularly translocated. Consequently, the NpAS yields were conducted to evaluate using shake-flask fermentation experiments, and the intracellular and extracellular activities of NpAS were assayed and summarized in Fig. 3b. After cultivation for 84 hr, the extracellular activity of NpAS reached 428.21 u/g (DCW), which was about 62.81% [428.21/(428.21 + 253.52)] of the total activity of NpAS expressed (Fig. 3b). The post-fermentation data suggested that NpAS peptides could be rapidly well-folded intracellularly and translocated extracellularly by *B. licheniformis* TAT pathway rather than Sec pathway.

**Fig. 4.**
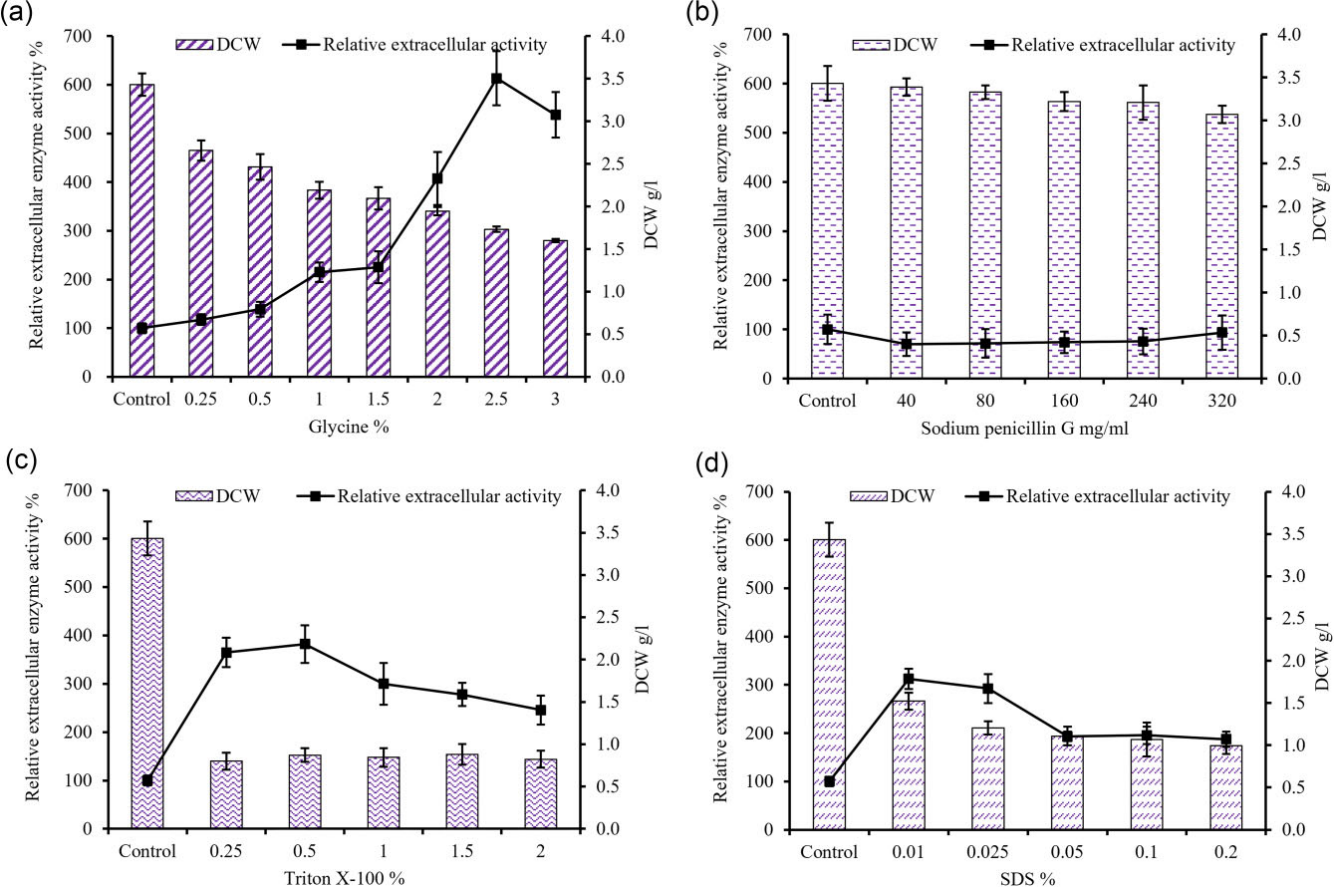
Comparison of DCW and NpAS relative production with different Glycine% (a), Sodium penicillin G mg/ml (b), Triton X-100% (c), and SDS% (d). The error bar indicates the standard deviation from three parallel experiments.

Studies have shown that altering the permeability of the cell wall can significantly enhance the secretion efficiency of recombinant proteins (Zhao et al., 2017; Yang et al., 2022). Consequently, we investigated the impact of various cell wall/membrane-weakening chemicals on the extracellular secretion of NpAS. Our findings indicated a considerable increase in extracellular NpAS when glycine was added, with the activity reaching approximately 6.13 times that of the control when 2.5% glycine was included (Fig. 4a). The enhanced secretion of NpAS may be attributed to increased cell permeability as glycine replaces L-alanine and D-alanine in the peptidoglycan of the cell wall during ongoing cell growth, resulting in a significantly weakened cell wall (Hammes et al., 1973). Other chemicals, such as sodium penicillin G, SDS, and Triton X-100, had little or negative effects on enzyme production (Fig. 4b–D). Specifically, sodium penicillin G was found to negatively impact NpAS yield (Fig. 4b). The addition of SDS and Triton X-100 significantly affected the dry cell weight, with extracellular activity increasing to about 3.82-fold (Fig. 4c) and 3.13-fold (Fig. 4d) more than the control when 0.5% Triton X-100 and 0.01% SDS were added, respectively. These results suggest that an optimal amount of glycine in the culture medium is more conducive to the secretion level of NpAS.

**Fig. 5.**
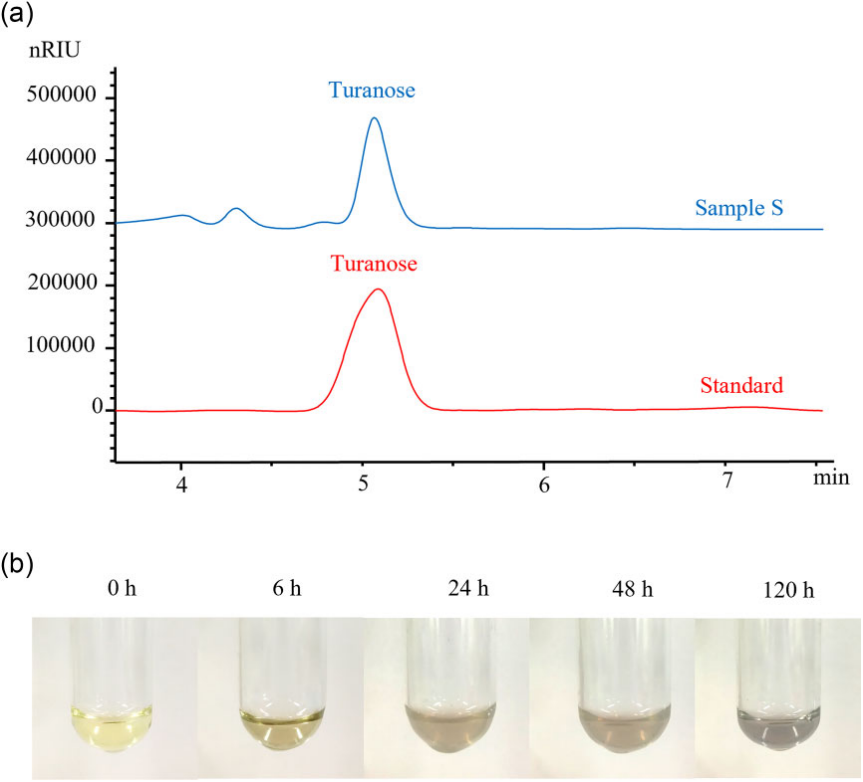
Characteristic of the products synthesized by NpAS. (a) HPLC profile of the reaction medium supernatant after 120 hr reaction in the presence of 75 mM sucrose (sample S). Standard is turanose. (b) The water-insoluble solution was stained with a dilute iodine solution. A total of 400 μl of each reaction mixture contained 75 mM sucrose and 0.1 u/ml of the enzymes in 50 mM Tris-HCl buffer (pH 7.0) and incubated at 35°C for 6 hr, 24 hr, 48 hr, and 120 hr.

To further check if the recombinant NpAS retained the same unique functions as that of the wild-type NpAS, extracellular NpAS from strain BL-1.0AS was prepared and purified. The formation of turanose and α-1,4-glucans (amylose) from sucrose by purified NpAS were examined. The resulting products were determined by using HPLC and iodine staining methods. Turanose, a specific product from sucrose through the isomerization activity of NpAS, was identified (Fig. 5a). α-1,4-Glucans, another specific product from sucrose through its transglucosylase activity were detectable at 6 hr and gradually increased over the course of the reaction (Fig. 5b). The results clearly indicated that recombinant NpAS expressed in *B. licheniformis* via the TAT pathway preserves the unique characters of the wild NpAS in synthesizing amylose type polymers and turanose (Wang et al., 2012; De Montalk et al., 2000).

The research results conclusively showed that NpAS could be expressed and secreted extracellularly in *B. licheniformis* via the TAT pathway. It was also demonstrated that the fetal peptide of the expressed NpAS in *B. licheniformis* might fold concurrently. It would be a plausible explanation that only the TAT pathway but not the Sec pathway in *B. licheniformis* can effectively transport the expressed NpAS (Berks, 2015), although a relatively high percentage of NpAS activity remained intracellularly. Enhancing the yield and translocation efficiency of NpAS in *B. licheniformis* through TAT pathway engineering is feasible, as will be detailed in subsequent studies.

## Conclusion

In this study, the new expression strategy was developed successfully for the secretory expression of NpAS in *B. licheniformis*. Neisseria polysaccharea amylosucrase, when expressed in *B. licheniformis*, is likely to fold rapidly and be translocated extracellularly via the TAT pathway rather than the Sec pathway. This innovative expression system for NpAS lays the groundwork for its large-scale and efficient preparation.

## References

[bib1] Albiniak A. M., Matos C. F., Branston S. D., Freedman R. B., Keshavarz-Moore E., Robinson C. (2013). High-level secretion of a recombinant protein to the culture medium with a *Bacillus subtilis* twin-arginine translocation system in *Escherichia coli*. FEBS Journal, 280(16), 3810–3821. 10.1111/febs.12376 23745597

[bib2] Anne J., Economou A., Bernaerts K. (2017). Protein secretion in gram-positive bacteria: From multiple pathways to biotechnology. Current Topics in Microbiology and Immunology, 404, 267–308. 10.1007/82_2016_49 27885530

[bib3] Arauzo-Aguilera K., Saaranen M. J., Robinson C., Ruddock L. W. (2023). Highly efficient export of a disulfide-bonded protein to the periplasm and medium by the Tat pathway using CyDisCo in *Escherichia coli*. MicrobiologyOpen, 12(2), e1350. 10.1002/mbo3.1350 37186227 PMC9995818

[bib4] Berks B. C. (2015). The twin-arginine protein translocation pathway. Annual Review of Biochemistry, 84(1), 843–864. 10.1146/annurev-biochem-060614-034251 25494301

[bib5] Bradford M. M. (1976). A rapid and sensitive method for the quantitation of microgram quantities of protein utilizing the principle of protein-dye binding. Analytical Biochemistry, 72(1–2), 248–254. 10.1016/0003-2697(76)90527-3 942051

[bib6] Büttcher V., Welsh T., Willmitzer L., Kossmann J. (1997). Cloning and characterization of the gene for amylosucrase from *Neisseria polysaccharea*: Production of a linear α-1,4-glucan. Journal of Bacteriology, 179(10), 3324–3330. 10.1128/jb.179.10.3324-3330.1997 9150231 PMC179114

[bib7] Choi S. W., Lee J. A., Yoo S. H. (2019). Sucrose-based biosynthetic process for chain-length-defined alpha-glucan and functional sweetener by *bifidobacterium* amylosucrase. Carbohydrate Polymers, 205, 581–588. 10.1016/j.carbpol.2018.10.064 30446144

[bib8] De Montalk G. P., Remaud-Simeon M., Willemot R. M., Sarcabal P., Planchot V., Monsan P. (2000). Amylosucrase from *Neisseria polysaccharea*: Novel catalytic properties. FEBS Letters, 471(2–3), 219–223. 10.1016/S0014-5793(00)01406-X 10767427

[bib9] Denks K., Vogt A., Sachelaru I., Petriman N. A., Kudva R., Koch H. G. (2014). The sec translocon mediated protein transport in prokaryotes and eukaryotes. Molecular Membrane Biology, 31(2–3), 58–84. 10.3109/09687688.2014.907455 24762201

[bib10] Ding H. Y., Wang T. Y., Wu J. Y., Tsai Y. L., Chang T. S. (2022). Enzymatic synthesis of novel and highly soluble puerarin glucoside by *Deinococcus geothermalis* amylosucrase. Molecules, 27(13), 4074. 10.3390/molecules27134074 35807322 PMC9268652

[bib11] Emond S., Mondeil S., Jaziri K., Andre I., Monsan P., Remaud-Simeon M., Potocki-Veronese G. (2008). Cloning, purification and characterization of a thermostable amylosucrase from *Deinococcus geothermalis*. FEMS Microbiology Letters, 285(1), 25–32. 10.1111/j.1574-6968.2008.01204.x 18522649

[bib12] Ha S. J., Seo D. H., Jung J. H., Cha J., Kim T. J., Kim Y. W., Park C. S. (2009). Molecular cloning and functional expression of a new amylosucrase from *Alteromonas macleodii*. Bioscience, Biotechnology, and Biochemistry, 73(7), 1505–1512. 10.1271/bbb.80891 19584557

[bib13] Hammes W., Schleifer K. H., Kandler O. (1973). Mode of action of glycine on the biosynthesis of peptidoglycan. Journal of Bacteriology, 116(2), 1029–1053. 10.1128/jb.116.2.1029-1053.1973 4200845 PMC285483

[bib14] He C. J., Yang Y. R., Zhao R. F., Qu J. Y., Jin L., Lu L. L., Xu L., Xiao M. (2018). Rational designed mutagenesis of levansucrase from *Bacillus licheniformis* 8-37-0-1 for product specificity study. Applied Microbiology and Biotechnology, 102(7), 3217–3228. 10.1007/s00253-018-8854-3 29497794

[bib15] Hehre E. J., Hamilton D. M. (1948). The conversion of sucrose to a polysaccharide of the starch-glycogen class by *Neisseria* from the pharynx. Journal of Bacteriology, 55(2), 197–208. 10.1128/jb.55.2.197-208.1948 16561448 PMC518429

[bib16] Jung D. H., Jung J. H., Seo D. H., Ha S. J., Kweon D. K., Park C. S. (2013). One-pot bioconversion of sucrose to trehalose using enzymatic sequential reactions in combined cross-linked enzyme aggregates. Bioresource Technology, 130, 801–804. 23357588 10.1016/j.biortech.2012.12.162

[bib17] Kim E. R., Rha C. S., Jung Y. S., Choi J. M., Kim G. T., Jung D. H., Kim T. J., Seo D. H., Kim D. O., Park C. S. (2019). Enzymatic modification of daidzin using heterologously expressed amylosucrase in *Bacillus subtilis*. Food Science and Biotechnology, 28(1), 165–174. 10.1007/s10068-018-0453-7 30815307 PMC6365322

[bib18] Kim K. H., Park Y. D., Park H., Moon K. O., Ha K. T., Baek N. I., Park C. S., Joo M., Cha J. (2014). Synthesis and biological evaluation of a novel baicalein glycoside as an anti-inflammatory agent. European Journal of Pharmacology, 744, 147–156. 10.1016/j.ejphar.2014.10.013 25446915

[bib19] Klaewkla M., Pichyangkura R., Charoenwongpaiboon T., Wangpaiboon K., Chunsrivirot S. (2020). Computational design of oligosaccharide producing levansucrase from *Bacillus licheniformis* RN-01 to improve its thermostability for production of levan-type fructooligosaccharides from sucrose. International Journal of Biological Macromolecules, 160, 252–263. 10.1016/j.ijbiomac.2020.05.102 32439436

[bib20] Lee D., Park S. D., Jun S. J., Park J. T., Chang P. S., Yoo S. H. (2022). Differentiated structure of synthetic glycogen-like particle by the combined action of glycogen branching enzymes and amylosucrase. International Journal of Biological Macromolecules, 195, 152–162. 10.1016/j.ijbiomac.2021.11.153 34856217

[bib21] Liu C. H., Lu J., Lu L. L., Liu Y. H., Wang F. S., Xiao M. (2010). Isolation, structural characterization and immunological activity of an exopolysaccharide produced by *Bacillus licheniformis* 8-37-0-1. Bioresource Technology, 101(14), 5528–5533. 10.1016/j.biortech.2010.01.151 20199860

[bib22] Moon K., Lee S., Park H., Cha J. (2021). Enzymatic synthesis of resveratrol α-glucoside by amylosucrase of *Deinococcus geothermalis*. Journal of Microbiology and Biotechnology, 31(12), 1692–1700. 10.4014/jmb.2108.08034 34584041 PMC9706033

[bib23] Moulis C., Andre I., Remaud-Simeon M. (2016). GH13 amylosucrases and GH70 branching sucrases, atypical enzymes in their respective families. Cellular and Molecular Life Sciences, 73(14), 2661–2679. 10.1007/s00018-016-2244-8 27141938 PMC11108324

[bib24] Nakapong S., Pichyangkura R., Ito K., Iizuka M., Pongsawasdi P. (2013). High expression level of levansucrase from *Bacillus licheniformis* RN-01 and synthesis of levan nanoparticles. International Journal of Biological Macromolecules, 54, 30–36. 10.1016/j.ijbiomac.2012.11.017 23219733

[bib25] Niu D. D., Cong H. H., Zhang Y. N., McHunu N. P., Wang Z. X. (2022). Pullulanase with high temperature and low pH optima improved starch saccharification efficiency. Scientific Reports, 12(1), 21942. 10.1038/s41598-022-26410-9 36536070 PMC9763405

[bib26] Niu D. D., Li C. Y., Wang P., Huang L., McHunu N. P., Singh S., Prior B. A., Ye X. Y. (2019). Twin-arginine signal peptide of *Bacillus licheniformis* GlmU efficiently mediated secretory expression of protein glutaminase. Electronic Journal of Biotechnology, 42, 49–55. 10.1016/j.ejbt.2019.10.006

[bib27] Niu D. D., Wang Z. X. (2007). Development of a pair of bifunctional expression vectors for *Escherichia coli* and *Bacillus licheniformis*. Journal of Industrial Microbiology and Biotechnology, 34(5), 357–362. 10.1007/s10295-007-0204-x 17256153

[bib28] Niu D. D., Zuo Z. R., Shi G. Y., Wang Z. X. (2009). High yield recombinant thermostable α-amylase production using an improved *Bacillus licheniformis* system. Microbial Cell Factories, 8(1), 58. 10.1186/1475-2859-8-58 19878591 PMC2776586

[bib29] Park M. O., Lee B. H., Lim E., Lim J. Y., Kim Y., Park C. S., Lee H. G., Kang H. K., Yoo S. H. (2016). Enzymatic process for high-yield turanose production and its potential property as an adipogenesis regulator. Journal of Agricultural and Food Chemistry, 64(23), 4758–4764. 10.1021/acs.jafc.5b05849 27253611

[bib30] Perez-Cenci M., Salerno G. L. (2014). Functional characterization of *Synechococcus* amylosucrase and fructokinase encoding genes discovers two novel actors on the stage of cyanobacterial sucrose metabolism. Plant Science, 224, 95–102. 10.1016/j.plantsci.2014.04.003 24908510

[bib31] Pizzut-Serin S., Potocki-Veronese G., van der Veen B. A., Albenne C., Monsan P., Remaud-Simeon M. (2005). Characterisation of a novel amylosucrase from *Deinococcus radiodurans*. FEBS Letters, 579(6), 1405–1410. 10.1016/j.febslet.2004.12.097 15733849

[bib32] Potocki-Veronese G., Putaux J. L., Dupeyre D., Albenne C., Remaud-Simeon M., Monsan P., Buleon A. (2005). Amylose synthesized *in vitro* by amylosucrase: Morphology, structure, and properties. Biomacromolecules, 6(2), 1000–1011. 10.1021/bm049326g 15762671

[bib33] Putaux J. L., Potocki-Veronese G., Remaud-Simeon M., Buleon A. (2006). α-D-Glucan-based dendritic nanoparticles prepared by *in vitro* enzymatic chain extension of glycogen. Biomacromolecules, 7(6), 1720–1728. 10.1021/bm050988v 16768390

[bib34] Putseys J. A., Lamberts L., Delcour J. A. (2010). Amylose-inclusion complexes: Formation, identity and physico-chemical properties. Journal of Cereal Science, 51(3), 238–247. 10.1016/j.jcs.2010.01.011

[bib35] Rha C. S., Kim H. G., Baek N. I., Kim D. O., Park C. S. (2020a). Amylosucrase from *Deinococcus geothermalis* can be modulated under different reaction conditions to produce novel quercetin 4'-O-α-D-isomaltoside. Enzyme and Microbial Technology, 141, 109648. 10.1016/j.enzmictec.2020.109648 33051009

[bib36] Rha C. S., Kim H. G., Baek N. I., Kim D. O., Park C. S. (2020b). Using amylosucrase for the controlled synthesis of novel isoquercitrin glycosides with different glycosidic linkages. Journal of Agricultural and Food Chemistry, 68(47), 13798–13805. 10.1021/acs.jafc.0c05625 33175543

[bib37] Riou J. Y., Guibourdenche M., Popoff M. Y. (1983). A new taxon in the genus *Neisseria*. Annals of Microbiology, 134B(2), 257–267. 10.1016/S0769-2609(83)80038-6 6651122

[bib38] Sambrook J., Russel D. (2001); Molecular Cloning: a Laboratory Manual, (3rd Ed.). Cold Spring Harbor Press.

[bib39] Schneider J., Fricke C., Overwin H., Hofer B. (2011). High level expression of a recombinant amylosucrase gene and selected properties of the enzyme. Applied Microbiology and Biotechnology, 89(6), 1821–1829. 10.1007/s00253-010-3000-x 21113589

[bib40] Seo D. H., Jung J. H., Choi H. C., Cho H. K., Kim H. H., Ha S. J., Yoo S. H., Cha J., Park C. S. (2012). Functional expression of amylosucrase, a glucan-synthesizing enzyme, from *Arthrobacter chlorophenolicus* A6. Journal of Microbiology and Biotechnology, 22(9), 1253–1257. 10.4014/jmb.1201.01056 22814500

[bib41] Seo D. H., Yoo S. H., Choi S. J., Kim Y. R., Park C. S. (2020). Versatile biotechnological applications of amylosucrase, a novel glucosyltransferase. Food Science and Biotechnology, 29(1), 1–16. 10.1007/s10068-019-00686-6 31976122 PMC6949346

[bib42] Seung D. (2020). Amylose in starch: Towards an understanding of biosynthesis, structure and function. New Phytologist, 228(5), 1490–1504. 10.1111/nph.16858 32767769

[bib43] Shen P. L., Niu D. D., Liu X. L., Tian K. M., Permaul K., Singh S., Mchunu N. P., Wang Z. X. (2022). High-efficiency chromosomal integrative amplification strategy for overexpressing alpha-amylase in *Bacillus licheniformis*. Journal of Industrial Microbiology and Biotechnology, 49(3), kuac009. 10.1093/jimb/kuac009 35325171 PMC9142198

[bib44] Tsirigotaki A., De Geyter J., Sostaric N., Economou A., Karamanou S. (2017). Protein export through the bacterial Sec pathway. Nature Reviews Microbiology, 15(1), 21–36. 10.1038/nrmicro.2016.161 27890920

[bib45] Valimets S., Pedetti P., Virginia L. J., Hoang M. N., Sauer M., Peterbauer C. (2023). Secretory expression of recombinant small laccase genes in gram-positive bacteria. Microbial Cell Factories, 22(1), 72. 10.1186/s12934-023-02075-5 37062846 PMC10108450

[bib46] van der Veen B. A., Skov L. K., Potocki-Veronese G., Gajhede M., Monsan P., Remaud-Simeon M. (2006). Increased amylosucrase activity and specificity, and identification of regions important for activity, specificity and stability through molecular evolution. FEBS Journal, 273(4), 673–681. 10.1111/j.1742-4658.2005.05076.x 16441655

[bib47] Wang R., Bae J. S., Kim J. H., Kim B. S., Yoon S. H., Park C. S., Yoo S. H. (2012). Development of an efficient bioprocess for turanose production by sucrose isomerisation reaction of amylosucrase. Food Chemistry, 132(2), 773–779. 10.1016/j.foodchem.2011.11.035

[bib48] Wang Y. C., Xu W., Bai Y. X., Zhang T., Jiang B., Mu W. M. (2017). Identification of an α-(1,4)-glucan-synthesizing amylosucrase from *Cellulomonas carboniz* T26. Journal of Agricultural and Food Chemistry, 65(10), 2110–2119. 10.1021/acs.jafc.6b05667 28240031

[bib49] Wu J. Y., Ding H. Y., Luo S. Y., Wang T. Y., Tsai Y. L., Chang T. S. (2022). Novel glycosylation by amylosucrase to produce glycoside anomers. Biology (Basel), 11(6), 822. 10.3390/biology11060822 35741343 PMC9220500

[bib50] Yang H. Q., Wang H. K., Wang F. X., Zhang K. J., Qu J. F., Guan J. M., Shen W., Cao Y., Xia Y. Y., Chen X. (2022). Efficient extracellular production of recombinant proteins in *E. coli* via enhancing expression of *dacA* on the genome. Journal of Industrial Microbiology and Biotechnology, 49(4), kuac016. 10.1093/jimb/kuac016 35648451 PMC9338883

[bib51] Zhao L., Ye J., Fu J., Chen G. Q. (2017). Engineering peptidoglycan degradation related genes of *Bacillus subtilis* for better fermentation processes. Bioresource Technology, 248(Pt A), 238–247. 10.1016/j.biortech.2017.05.134 28811162

[bib52] Zhou C., Zhou H., Li D., Zhang H., Wang H., Lu F. (2020). Optimized expression and enhanced production of alkaline protease by genetically modified *Bacillus licheniformis* 2709. Microbial Cell Factories, 19(1), 45. 10.1186/s12934-020-01307-2 32093734 PMC7041084

[bib53] Zhu G. J., Wang Z. X. (1994). Industrial Microbiology: a Laboratory Manual. China Light Industry Press.

